# Natural killer cells in multiple sclerosis: foe or friends?

**DOI:** 10.3389/fncel.2025.1500770

**Published:** 2025-04-04

**Authors:** Fatemeh Aghaee, Mohammadreza Abedinpour, Saeid Anvari, Alia Saberi, Amir Fallah, Arash Bakhshi

**Affiliations:** ^1^Member Research Committee, School of Medicine, Guilan University of Medical Sciences, Rasht, Iran; ^2^Department of Neurology, Neurosciences Research Center, Poursina Hospital, School of Medicine, Guilan University of Medical Sciences, Rasht, Iran; ^3^Department of Internal Medicine, Regenerative Medicine Research Center, Razi Hospital, School of Medicine, Guilan University of Medical Sciences, Rasht, Iran

**Keywords:** multiple sclerosis, natural killer, inflammation, immunity, protection

## Abstract

Multiple sclerosis (MS) is an immune-mediated disorder involving the central nervous system (CNS), in which demyelination is caused. The initiation and progression of MS is thought to depend largely on CD4^+^ T lymphocytes, yet new data has emphasized the involvement of the innate immune system in the MS disease responses. Generally, several types of immune cells play a part, with natural killer (NK) cells being essential. Different subsets of natural killer cells function differently within the course of an autoimmune disease, such as MS. There are mainly two types of natural killers in humans: immature CD56^*bright*^ CD16^–^ and mature CD56^*dim*^ CD16^+^ natural killers, together with their respective subtypes. Factors from natural killers expand the T cell population and control the process by which native CD4^+^ T cells differentiate into Th1 or Th2 lymphocytes, which affect autoimmune responses. Natural killer subsets CD56^*bright*^ and CD56^*dim*^ may have differing roles in MS development. The impact of these NK cell subsets is influenced by factors such as Granzymes, genetics, infections, TLR, and HSP. We reviewed and evaluated the relationship between natural killer cells and MS.

## 1 Introduction

The leading contributor to neurological disability in young people is multiple sclerosis (Mastorodemos et al., 2015). It refers to an immunological disorder affecting the CNS and involves inflammatory and demyelinating processes (Oh et al., 2018; Assari, 2020). Multiple sclerosis can affect any central nervous system component, leading to various disease manifestations (Doshi and Chataway, 2017). Both adaptive and innate immune systems are involved in MS pathogenesis (Liu et al., 2021). Different subsets of Natural killer cells function differently within the course of an autoimmune disease, such as MS (Gianchecchi et al., 2021).

MS may be affected or improved by natural killers through mechanisms of immunoregulation (Esmaeil Amini et al., 2020; Ahmadi et al., 2022). These cells are the main producers of cytokines, including TNF-α, GM-CSF, and IFN-γ. There are two major types of natural killer cells in humans: CD56^*bright*^ and CD56^*dim*^ NK cells (Schwane et al., 2020). CD56, which is often present in natural killers, is considered an important marker of treatment outcome (Laroni and Uccelli, 2020). Previously, research demonstrated that natural killer cells carry out various functions, including stimulating tissue and cell growth, exerting antitumor and antiviral activities, and modulating and modulating inflammatory responses (Poznanski and Ashkar, 2019; Bakhshi et al., 2023). Under particular conditions, NK can modulate autoimmune activity in the CNS.

Natural killer cells are multifunctional innate immune cells that are instrumental in providing the host defense and regulating the immunity of the host. The involvement of NK lymphocytes in MS disease course and treatment outcome is suggested by evidence from both individuals with multiple sclerosis and experimental autoimmune encephalomyelitis (EAE), an animal model of MS. However, their exact role is disputed. The impact of NK cell functions on MS pathogenesis remains unclear since research has primarily focused on peripheral natural killer cells instead of local tissue-resident natural killer cells and reported inconsistent results (Mimpen et al., 2020; Dhaiban et al., 2021; Mi et al., 2021). In our review study, we investigate the relationship between NK lymphocytes and MS and the impact of different NK cell subsets (protective or pathologic) on MS progression.

## 2 Characteristics and general features of multiple sclerosis

In addition to sensory impairments, bladder malfunctions, mental impairments, limb weakness, and incoordination could vary based on the location of the pathological changes in the central nervous system (Sadeghi Hassanabadi et al., 2022). It is common to find lesions around the brain’s ventricles in white and gray matter. The interplay of various effectors and immunity-regulatory cells may influence inflammatory processes in the CNS. MS can be divided into three subtypes: relapse remitting MS (RRMS), primary progressive MS (PPMS), and secondary progressive MS (SPMS) (Bakhshi et al., 2023). Typically, multiple sclerosis manifests as flare-ups, and then intermittent clinical stability for the majority of patients, but 10% of people progress slowly and have no neurological recovery from the onset (Kaur et al., 2013). A transient inflammatory response of the CNS causes damage to myelin and axons, leading to neurological dysfunction in the early stages (Beliën et al., 2022). Even though MS has axonal degeneration at an early stage, inflammation in MS is more extensive and chronic than that of primary neurodegenerative disorders or acute brain damage (Hemmer et al., 2002). T lymphocytes are the essential component in the development of the neurodegenerative MS in the CNS. The involvement of the innate immune system in the MS disease reaction has been emphasized by recent studies (Calahorra et al., 2022).

Multiple sclerosis involves Th lymphocytes that are suspected to be pro-inflammatory, such as Th1, Th17, and GM-CSF-secreting effector cells, as well as follicular Th cells. These effector cell subsets are likely to show abnormal activity because CD4^+^ regulatory T lymphocytes lack normal function. CD4^+^ T cells capable of cytotoxicity are thought to be involved in the process of multiple sclerosis, whether by causing damage or by helping through the controlled elimination of other pro-inflammatory cells. Follicular Th lymphocytes are recognized by their simultaneous expression of CXCR5 and PD-1 and through the release of IL-21 and IL-4, which are essential for B lymphocyte survival, proliferation, and plasma cell differentiation (Ahmadi et al., 2022). Follicular Th lymphocytes can enhance Th17 and B lymphocyte reactions, thus increasing neuroinflammatory responses. Even though CD4^+^ T lymphocytes might have a significant influence on the peripheral immune processes that lead to multiple sclerosis, it has been known for a long time that CD8^+^ T lymphocytes are more prevalent than CD4^+^ T lymphocytes in the brain lesions of MS patients. CSF CD8^+^ T lymphocytes associated with multiple sclerosis have active and memory phenotypes and can release effector cytokines, including IFN-γ, TNFα, and IL-17. Understanding the pathogenesis of multiple sclerosis requires a basic understanding of the blood-brain barrier (BBB), the integrity of which is compromised by MS lesions (Høglund and Maghazachi, 2014; Romaniuc et al., 2020). B cells primarily contribute to CNS autoimmunity by producing antibodies that react against myelin and cause demyelination and axonal damage. They are also responsible for presenting myelin components to T cells, which leads to the production of antibodies and promotes autoreactivity (Mastorodemos et al., 2015).

T lymphocytes that are activated can invade the CNS parenchyma, causing inflammation by secreting cytokines, including IL-17 and Interferon gamma (IFN-γ). As a result of these pro-inflammatory cytokines, other leukocytes are attracted to the site, and there is direct damage to neural tissue (Galicia and Gommerman, 2014). When properly stimulated, human invariant natural killer (iNKT) cells produce pro-inflammatory and anti-inflammatory cytokines, indicating they are a powerful immune control mechanism. iNKT cells predominantly produce pro-inflammatory molecules, notably IFN-γ and Tumor Necrosis Factor-alpha (TNF-α) (Yamamura et al., 2007), regarding the microglial cells, and depending on the inflammatory environment in the CNS. They can release anti-inflammatory substances (TGF-ß and IL-10). It has been suggested that microglia contribute to neurogenesis because they secrete neurotrophins, including brain-derived neurotrophic factor (BDNF), insulin-like growth factor-1 (IGF-1), and Neurotrophin 3 (NT-3) (Gandhi et al., 2010).

## 3 Natural killer cell

### 3.1 NK cell subsets

Natural killer cells are essential to immunity as a front-line immune response against viral pathogens and neoplasms (Kaur et al., 2013). Further to the cytotoxic functions of natural killers, which are essential to the innate immune system, they are capable of suppressing T lymphocytes either through the elimination of antigen-presenting cells or by the release of immunosuppressive substances, including IL-10. Anti-inflammatory cytokines are abundantly produced by the NK cell subset, which performs a “regulatory” function (Cooper et al., 2001). The chemokines released by these cells may further promote the accumulation of other regulatory leukocytes, namely FoxP3^+^ regulatory NK cells (Segal, 2007). NK cells are derived from CD34+ hematopoietic stem cells (HSCs), undergoing distinct developmental phases marked by the progressive expression of surface receptors and functional maturation. These progenitor cells, including lineage-committed precursors, may migrate from the bone marrow to peripheral tissues, where terminal differentiation occurs. Early precursors are characterized by the absence of CD34 and the presence of CD117, CD244, and CD161, but lack CD56 expression (Stabile et al., 2015). As maturation progresses, CD56 is acquired, followed by sequential expression of activating receptors such as NKp46, NKG2D, and DNAM-1. This transition culminates in a phenotype resembling peripheral blood (PB) CD56^*bright*^ NK cells, which exhibit surface markers like NKp44 (Bozzano et al., 2015; Scoville et al., 2017).

Peripheral blood NK cells exist as heterogeneous populations, reflecting divergent differentiation states or functional specializations (Montaldo et al., 2013). The primary subsets are distinguished by surface density of CD56: CD56^*bright*^ and CD56^*dim*^ NK cells. Historically, CD56^*bright*^ cells were associated with cytokine secretion, whereas CD56^*dim*^ subsets were linked to cytotoxicity (Weber et al., 2024). Recent evidence, however, demonstrates that cytotoxic CD56^*dim*^ cells also secrete substantial cytokines upon receptor activation (Cooper et al., 2001). A large percentage of circulating and spleen natural killers consist of CD56^*dim*^ CD16^+^ NK cells. Upon interacting with tumor cells, these cytotoxic cells produce IFN-γ (Sadeghi Hassanabadi et al., 2022). When CD56^*bright*^ NK cells encounter a dangerous or cancerous cell, they create an inflammatory condition that helps to eliminate the cancerous cells. However, this pro-inflammatory condition can also worsen autoimmune disorders, such as MS, if the immune system is reactivated (Mimpen et al., 2020). Most NK cells in a lymph node and tonsil are CD56^*bright*^CD16^–^ NK cells and do not produce perforin; however, they release molecules, including IFN-γ, as a result of IL-12, 15, and 18 stimulation (Sadeghi Hassanabadi et al., 2022). CD56^*bright*^ NK cells act as immunoregulators by producing cytokines as a result of chemical signals. As well as producing granzyme K, CD56^*bright*^ NK cells eliminate active CD4^+^ T lymphocytes. Natural killers with high expression of CD56 antigen (CD56^*bright*^ NK cells) do not exhibit killer-cell immunoglobulin-like receptors (KIR) (Fehniger et al., 2003; Rodriguez-Mogeda et al., 2024). They exhibit heightened responsiveness to cytokine signaling via receptors like IL-2R and IL-15R but reduced sensitivity to direct cellular interactions (Mimpen et al., 2020).

Multiple subsets of NK cells are believed to play a role in immunoregulatory processes in MS, based on studies of peripheral blood and cerebrospinal fluid. Dendritic cells (DCs), pivotal initiators of immune responses, modulate NK cell activity through direct contact and release of soluble mediators, including type I interferons, IL-12, IL-15, and IL-18. Among these, IL-12 is critical for enhancing NK cell proliferation and interferon-γ production, and had long been regarded as the central mediator amplifying signaling pathways critical to natural killer cell effector capabilities (Lagumersindez-Denis et al., 2017). Natural killer cells have various surface markers, which allow different methods to classify them into different groups. CD56^*dim*^ NK cells have anti-tumor cytotoxic activity, while each group can produce cytokines. Both NK cell groups have NKp46- and NKp80-activating surface receptors. CD56^*bright*^ NK cells differentiate CD56^*dim*^ NK cells by the expression of CD16 and PEN5 (Roma, 2021; Chu et al., 2022). Human CD3^–^CD56^+^ NK subsets express low levels of CD8. The CD56^+^ CD8^*low*^ subset of human NK cells is capable of a greater degree of cytolytic activity than CD8^–^ subsets (Lowdell et al., 2002). Subsequently, this was expanded to include acute myeloid leukemia cases in complete remission following monotherapy chemotherapy (Addison et al., 2005). A second way to distinguish them is through their cytokine profiles, such as NK1 and NK2, which express IFN-γ and IL-10 (NK1) or IL-5 and IL-13 (NK2) (Lagumersindez-Denis et al., 2017). NK cells with CD56^*dim*^ subsets have primarily cytotoxic functions, whereas NK cells with CD56^*bright*^ subsets secrete an abundance of inflammatory cytokines that normally act as regulators (Campbell and Hasegawa, 2013). It is estimated that CD56^*dim*^ NK cells produce ten times the amount of perforin and granzyme A in their granules than their CD56^*bright*^ NK cells counterparts. So, CD56^*dim*^ NK cells could have a more remarkable cytolytic ability due to this feature. However, NK cells with CD56^*bright*^ NK cells surfaces secrete more immunological cytokines, including TNF-α, IFN-γ, and IL-10 (Jacobs et al., 2001).

### 3.2 NK Cell activator, inhibitor, and associated signaling pathway

Natural killer cells express numerous receptors that can activate their cytotoxic and secretory functions. These receptors identify ligands on the surface of infected, transformed, or stressed cells (NKG2D). Signaling through inhibitory receptors also equips NK cells with appropriate responsiveness via a process known as licensing (KIR) (Kumar, 2018). Different mechanisms are involved in regulating NK cell activation in order to minimize unwanted reactions. Initially, natural killer cells possess inhibitory receptors capable of recognizing a wide variety of ligands. In addition, upregulating host ligands for activating receptors may result in inadvertent damage (Fogel et al., 2013). The NK cell’s surface is coated with various germline-encoded inhibitory and activating receptors. HLA genes encode classic or nonclassical major histocompatibility complex (MHC) class I proteins, inhibiting NK cell function by binding to them (Paul and Lal, 2017). Some activated receptors recognize ligands similar to inhibitory receptors, and some identify molecules with a structure consistent with MHC class I molecules, which have been increased by cellular stress. Human tissues normally have low levels of MICA and MICB, yet these molecules are expressed at higher levels in epithelial tumors and stressed cells. MHC-like molecules, including MICA and RAE-Iβ, that increase in expression under stress bind to and are detected by the activating receptor NKG2D (Deng and Mariuzza, 2006).

As natural killers detect ligands that are produced in response to stress on their targets via natural cytotoxicity receptors, distressed cells activate them (Long and Rajagopalan, 2002). Various inhibitory receptors are present in activated cells, as demonstrated in previous studies. One is the killer-cell immunoglobulin-like receptor family (KIR), which interacts with HLA-I. Without the presence of “self” ligands, natural killers activate and kill their targets as a result (Pende et al., 2019). KIR receptors provide inhibitory and activating signals that determine NK cell responses, but cytokine stimulation also influences NK cell activation thresholds (Long et al., 2013). Ligands that are widely distributed on the host’s membrane, called MHC class I, prevent natural killer cells from responding. Nevertheless, a lower activation threshold can be observed when infection is present (Le Maux Chansac et al., 2005; Zucchini et al., 2008). Consequently, the increased production of natural killer triggers on host cells is minimized when cellular stress or infection is absent (Frank and Paust, 2020).

## 4 Demographic and immunological effects on NK cells in MS patients

### 4.1 Age and NK cell

As individuals age, their immune cell subpopulations undergo alterations in composition (Table 1). Immunosenescence refers to age-associated alterations in immune function that elevate susceptibility to age-related diseases and mortality risk in elderly populations. This progressive deterioration of immune competence alters key immunological functions, including dysregulation of inflammatory responses, impaired antimicrobial defenses, and heightened predisposition to autoimmune conditions such as multiple sclerosis (Dema et al., 2025). Age-associated modifications in NK cell populations and functional capacities may indirectly modulate MS pathogenesis.

**TABLE 1:** The effect of demographic and immunological factors on NK cells in MS patients.

Variable	CD56 dim	CD56 bright	Description	*P*-value	References
TLR	Comparison of TLR expression in dim and bright NK				
TLR-2	98	96	Unit: percentage	NA	Deeba et al., 2020
TLR-3	60	38		NA	
TLR-7	65	60		NA	
TLR-9	70	60		NA	
**Comparison of NK dim and bright in different type of MS disease**					
RRMS	14.6	17.6	Absolute number of NK in CSF	NA	Rodríguez-Martín et al., 2015
RRMS	90	10	Percentage (%) of NK cells in DMD treated patients	*P* < 0.05	Martínez-Rodríguez et al., 2016
RRMS	40	4	Percentage (%) of NK cells in DMD treated patients	*P* < 0.05	Tahrali et al., 2018
RRMS	50.3	7.8	Unit: 109/L (count)	NA	Martínez-Rodríguez et al., 2011
RRMS	10	1	Unit: percentage of total lymphocytes	NA	Gross et al., 2016
SPMS	99	1	Percentage (%) of NK cells	*P* > 0.05	Plantone et al., 2013
PPMS	98	2		*P* > 0.05	
Granzyme	Comparison of Granzyme and perforin expression in dim and bright NK				
Granzyme A	9,000	8,000	Unit: MFI	*P* > 0.05	Plantone et al., 2013; Huth et al., 2016
Granzyme A	78	29	Unit: Pg/mL	NA	
Granzyme B	3,000	1,200	Unit: MFI	*P* > 0.05	
Granzyme B	65.5	11	Unit: Pg/mL	NA	
Granzyme K	1,500	4,500	Unit: MFI	*P* < 0.05	
Perforin	51.66	1.4	Unit: Pg/mL	NA	
Cytokine	Comparison of cytokine expression in dim and bright NK				
IL-17	4	3	Unit: ng/mL	NA	Sasson, 2009; Hao et al., 2011; Tahrali et al., 2018
IL-7	200	400	Unit: pg/mL	*P* < 0.05	
IFN-y	2	4	Unit: ng/mL	NA	
IFN-y	60	110	Unit: MFI	*P* < 0.05	
IL_10	0.3	1	Unit: ng/mL	NA	Tahrali et al., 2018
IL-22	0.5	1.5	Unit: ng/mL	NA	
Genes	Comparison of gene expression in dim and bright NK				
Bcl-2	20	900	Unit: copy/mL	*P* < 0.05	Sasson, 2009
Treatment	Comparison of NK dim and bright in different treatments				
NAT	90	10	Unit: Percentage	*P* < 0.05	Martínez-Rodríguez et al., 2011; Mellergård et al., 2013; Caruana et al., 2017; Acar et al., 2020
NAT	92	8	Unit: Percentage	NA	
Fing	95	5	Unit: Percentage	*P* > 0.05	
GA	90	10	Unit: Percentage	*P* > 0.05	
IFN	85	15	Unit: Percentage	*P* < 0.05	
IFN	170	20	Unit: Cell/μL	*P* < 0.05	
IFN	200	40	Unit: Cell/μL	NA	

The aging process, along with the proportion of different types of NK cells, possibly impacts the activity, secretion, and cytotoxicity of NK cells (Yan et al., 2010). This decrease in binding can be ascribed to an age-related impairment in secreting perforin, which can be attributed to an impaired alignment of lytic granules toward the immunological synapse (Hazeldine et al., 2012). The impact of aging on the production of cytokines and chemokines by CD56 NK cells remains unclear (Lutz et al., 2011). However, it can be speculated that decreased expression of CD94 may lead to more rigorously regulated NK cell activity and potentially a reduction in NK cell killing (Martínez-Rodríguez et al., 2010). In contrast, distinct activating receptors elicit the release of cytokines (such as IFN-γ and TNF-α). In the context of NK cell receptors, which can be either inhibitory or activating, the expression of certain receptors is influenced by aging (Fauriat et al., 2007). This is consistent with previous research that has demonstrated a reduction in CD56^*bright*^ NK cells and an increase in CD56^*dim*^ NK cells in healthy aging. Consequently, older subjects have a higher ratio of CD56^*dim*^ to CD56^*bright*^ NK cells (Kutzelnigg et al., 2005).

The precise role of NK cells in aging and their impact on MS remain incompletely understood. However, age-related shifts in NK cell subpopulations provide a basis for hypothesizing potential mechanisms linking Immunosenescence to MS pathophysiology. Age-associated declines in perforin secretion capacity, reduced CD94 receptor expression, and diminished cytotoxic activity of NK cells may attenuate neuroinflammation and decelerate autoimmune processes in MS. Conversely, decreased frequencies of s CD56^*bright*^ NK cells might compromise their immunoregulatory functions, potentially exacerbating MS progression. While these theoretical pathways offer plausible explanations, rigorous experimental and clinical studies are necessary to elucidate the complex interplay between NK cell aging and MS pathophysiology.

### 4.2 NK cell frequency and rolls in MS types

Various subsets of NK cells appear to play varying roles in MS initiation or progress. CD56^*dim*^ NK cells exhibit cytotoxic activity, whereas CD56^*bright*^ NK cells release substantial quantities of anti-inflammatory cytokines, contributing to their modulatory activity (Cooper et al., 2001). The total number of NK cells in circulation was lower in untreated RR-MS patients in comparison with both healthy individuals and treated RRMS participants (Gayoso et al., 2011). Specifically, the subset of CD3^–^CD16^–^ CD56^*bright*^ NK cells showed a significant increase in treated RR-MS cases in comparison with healthy individuals and untreated RRMS patients. The production of IL-10 by the CD3^–^CD16^–^CD56^*bright*^ NK cells was increased in treated RRMS individuals (Tahrali et al., 2018).

A higher percentage of CD3^–^CD56^*dim*^ NK cells that produce perforin is observed in patients with PPMS and SPMS as opposed to those without these conditions, suggesting that this particular group is involved in the progression of these types of MS. These forms are characterized by a continuous progressing condition and more widespread damage to the axons, even in inactive lesions and normal areas of white matter (Ruder et al., 2021). Notably, no significant disparities were observed in the proportions of circulating CD3^–^CD56^*dim*^ NK cells perforin+ natural killers between PPMS and SPMS participants. In an investigation comparing individuals diagnosed with PPMS and SPMS during remission phases to healthy subjects, Plantone and colleagues observed that the higher percentage of peripheral perforin-expressing CD56^*dim*^ NK cells in PPMS and SPMS cases indicates that they may contribute to developing these MS forms. Additionally, the proportion of CD3^–^CD56^*bright*^ NK cells showed a negative correlation with age in individuals with PPMS, while the proportion of CD3^–^CD56^*dim*^ NK cells showed a positive correlation with age (Plantone et al., 2013). Previous studies have also reported a natural decline in the CD3^–^ CD56^*bright*^ NK cells subset and an increase in the CD3^–^ CD56^*dim*^ NK cells subset with healthy aging (Gayoso et al., 2011).

When considering their ratio, it appears that the proportion of natural killer cells and their subpopulations relative to CD4^+^ T cells and IL-17A^+^ CD4^+^ T lymphocytes play a significant role in determining pathogenic activity. In a study examining the clinical significance of immune cell ratios in multiple sclerosis, Mimpen and colleagues investigated NK cell and T lymphocyte subpopulations in peripheral blood samples to assess their association with disease progression. The analysis included baseline peripheral blood mononuclear cell specimens collected from 50 participants with relapsing-remitting multiple sclerosis. Researchers identified significantly reduced proportions of NK cells relative to CD4^+^ T lymphocytes, as well as diminished CD56^*dim*^ NK cell-to-CD4^+^ T cell ratios, among patients demonstrating MRI-detectable inflammatory lesions. These findings propose that quantifying NK cell and CD4^+^ T lymphocyte balance could serve as a predictive biomarker for identifying subclinical or active neuroinflammatory processes in MS (Mimpen et al., 2021). However, further investigation using additional datasets from separate sources is necessary to validate the accuracy and usefulness of NK/CD4^+^ subpopulation ratios as indicators of prognosis in RRMS.

Through the phenotypic analysis of CD56^*dim*^ NK cells, it was found that patients in remission exhibited higher frequencies of cells expressing HLA-DR and CD54, while the frequency of CD11c-expressing NK CD56^*dim*^ NK cells was lower (Aranami et al., 2006). Recent findings suggest that remission RRMS patients, who are likely in an inactive disease state, have circulating CD56^*dim*^ NK cells characterized by an activated phenotype marked by HLA-DR expression. Interestingly, remission RRMS patients displayed increased frequencies of CD54^–^ expressing NK cells, indicating that NK cells may have the ability to migrate to sites of inflammation. For a more comprehensive understanding, it would be beneficial to investigate the expression of a broader range of molecules to better elucidate the functional characteristics of NK cells and their subpopulations (Darlington et al., 2018). Furthermore, additional research is needed to assess the direct involvement of CD56 NK cells in the detrimental effects on axons within MS lesions and their influence on disease activity across different forms of MS (Beliën et al., 2022).

**FIGURE 1:**
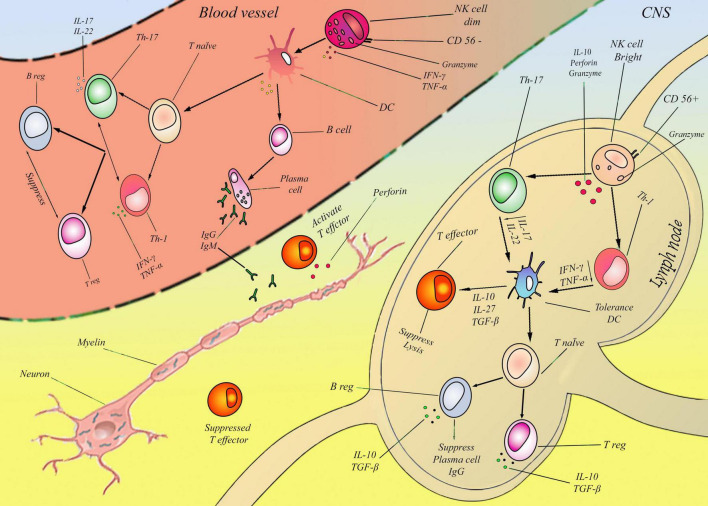
According to previous studies, myelin degeneration may be associated with NK cells in two ways: bright and dim NKs. These two cells’ effect on MS was proved, but their detailed mechanism is almost unknown. In this figure, a new mechanism is presented for two NK cells. Lymph node: In the first step CD56^*bright*^ NK cells produce Granzyme and IL-10 by default. IL-10 suppresses Th-1,17 and subsequently reduces IFN-γ, TNF-α, and IL-17, IL-22 in the second step. Reduction of these cytokines tolerated DCs and induced IL-10, 27, and TGF-β in the third step. In the fourth step, in cellular immunity, the T effector and cell lysis mechanism are suppressed, and T native cells differentiate to T-reg, consequently inducing IL-10 and intensifying this cycle. Also, in humoral immunity, the plasma cell was suppressed and induced B-reg with TGF- β. In sum, CD56^*bright*^cells suppressed two mains humoral (Plasma cell/IgG) and cellular (effector T) pathways, mainly by IL-10 and TGF- β. Blood vessels: CD56^*dim*^ NK cells in the first step produce IFN-Y and TNF-a. Subsequently, in the second step, these two cytokines directly activated DCs and continuously affected cellular and humoral systems by inflammatory cytokines in the third step. In the cellular immune system, T-native cells differentiated to Th-1, 17 and induced the production of IFN-γ, TNF-α, and IL-17, IL-22. These factors activated T-effectors and suppressed T-reg cells. Also, in the humoral immunity system, B cells are converted to plasma cells and induced IgG and IgM. In sum, CD56^*dim*^ cells activated two main humoral (Plasma cell/IgG, IgM) and cellular (effector T) pathways.CD56^*dim*^ NK cells developed demyelination by activating auto-antibody and perforin from plasma cells and effector T cells, and CD56^*bright*^cells inhibit demyelination by suppressing plasma cells and effector T cells. It is important to emphasize that this figure only outlines the fundamental and overarching pathways involving natural killer (NK) cells in the context of multiple sclerosis (MS), while omitting various influential factors such as therapeutic interventions.

CD56^*bright*^ NK cells eliminate (autologous) active T lymphocytes, severely restricting T lymphocyte activity (Jiang et al., 2011). It appears that CD56^*bright*^ NK cells proliferate more rapidly than Treg. It proves how CD56^*bright*^ NK cells are immunoregulatory and protective in MS (Caruana et al., 2017). In addition to CD56^*bright*^ NK cells, CD56^*dim*^ NK cells eliminate autologous antigen-activated CD4^+^ T lymphocytes. A variety of stimulants activate natural killers to restrain T lymphocyte proliferation or to increase their cytotoxicity (Gross et al., 2016; Figure 1).

The CD56^*dim*^ CD16^+^ NK cells secretes type 1 cytokines like IFN-γ and TNF-α, while the CD56^*bright*^ CD16^–^ NK cells secretes type 2 cytokines like IL-4 and IL-5 (Takeyama et al., 2021). CD56^*bright*^ CD16^–^ NK cells produce Th2 cytokines, including IL-4, IL-5, and IL-13, and are abundant in Fas (CD95) (Chen and Wang, 2019). Anti-CD3 and anti-CD52 monoclonal antibodies co-stimulate the ex vivo growth of natural killer cells from human peripheral blood mononuclear cells. Additional data showed the modulating role of natural killer cells, which is linked to CD95 expression reduction before relapse (Takahashi et al., 2004). It has been shown that MS patients in remission have a higher CD56^*bright*^ CD16^–^ NK cell population than those with relapse, implying that this subpopulation is beneficial to the remission process. In addition, CD56^*bright*^ CD16^–^ NK cells seem to influence antigen-specific autoreactive T-cell activation negatively (Takahashi et al., 2004).

### 4.3 Infection and NK cell

Viral infections are among the main environmental risk elements associated with MS, and NK lymphocytes are essential for the host’s defense against them (Table 1). The role of NKG2C^+^ NK lymphocyte proliferation in MS, which may be caused by Human Cytomegalovirus (HCMV) infection or other immune effects, is unclear (Hammer et al., 2018). CMV may have a protective effect on MS pathogenesis due to its ability to configure the immune system (Waggoner et al., 2011). For instance, one hypothesis suggests that CMV infection generates memory-like natural killer cells that are very effective against herpesviruses, such as EBV. CMV infection also appears to cause various modifications in the immunity, especially in NK and T lymphocytes (Moreira et al., 2019). Some studies have shown that NK lymphocytes work as immunoregulators and that the NKG2C^+^ NK lymphocyte subpopulation could influence the adaptive immune processes involved in MS development (Picarda and Benedict, 2018). Thus, HCMV could have a positive impact on MS, reducing disability worsening risk in patients with NKG2C^+^ NK lymphocyte proliferation induced by the virus. In a study assessing the immunological effects of latent CMV infection in individuals with MS, Prii and colleagues conducted flow cytometric immunophenotyping of NK cells, CD8^+^ T lymphocytes, and NKT-like cells in peripheral blood samples. Their analyses revealed distinct phenotypic profiles in these immune subsets among CMV-seropositive MS patients, marked by elevated expression of the activating NKG2C surface receptor. Furthermore, higher NKG2C receptor density was positively correlated with elevated Expanded Disability Status Scale (EDSS) scores, suggesting a potential link between CMV-driven immune adaptations and neurological disability progression in MS (Perri et al., 2024).

NK lymphocytes may interact with EBV through several mechanisms. As key components of the innate immunity against viruses, NK cells participate in the initial defense against EBV infection. An increase of NK lymphocytes is observed in infectious mononucleosis, which is related to a lower count of viruses in some cases (Bakhshi et al., 2024). The infection also seems to induce phenotype alterations in NK lymphocytes, such as an increase of NKG2A expression but not a memory-like NK cell phenotype (Chijioke et al., 2013).

The heterogeneous clinical outcomes of HSV-1 (Herpes simplex virus) infections indicate that host genes affecting susceptibility play a role in its regulation. MS patients who have the KIR2DL2 gene showed reduced activation of NK lymphocytes and slower elimination of HSV-1, according to a prior investigation (Cassidy et al., 2015). KIR2DL2 and the subsequent SHP1-SHP2 signaling pathway impair the efficiency of NK lymphocytes in controlling HSV-1 development. In contrast, the lack of KIR2DL2 or the inhibition of the SHP1-SHP2 signaling pathway is thought to protect against viral infections by facilitating an immune response to counteract viral invasion. The first investigation of the cytokine profile changes due to KIR2DL2 expression on NK lymphocytes revealed distinct cytokine secretion in KIR2DL2^+^ MS patients. KIR2DL2^+^ NK lymphocytes from individuals with MS produced a significant amount of Th17 cytokines, primarily IL-17A, throughout the HSV-1 infection course (Rizzo et al., 2016; Hedayati-Ch et al., 2024). Conversely, MS patients’ KIR2DL2- NK lymphocytes and NK lymphocytes from both KIR2DL2^+^ and KIR2DL2^–^ control subjects secreted Th1 cytokines, mainly IFN-γ. Interestingly, the variation in cytokine release among individuals with multiple sclerosis does not appear to be correlated with a distinct distribution of CD56^*bright*^ CD56^*dim*^ NK lymphocyte subpopulations in MS subjects who possess the KIR2DL2 receptor and those who do not (Bortolotti et al., 2022).

Herpesviruses that may influence MS development have been shown to modify NK phenotype, indicating the relevance of their phenotypes in predicting specific pathogens infections in MS. EBV infection could promote the expansion of early differentiated NK cells (CD56^*dim*^ CD57^–^) (Azzi et al., 2014).

### 4.4 HSP and NK cells

We demonstrated in a previous paper that the development of EAE was inhibited by pretreatment of mice with Hsp70 (Heat shock protein) complexed with CNS tissue-isolated non-characterized peptides and that this mechanism hinged on NK lymphocyte activity. In addition, the immunoregulation of EAE resulted from alterations in Dendritic Cell (DC) antigen presentation, which was caused by interactions involving Hsp70–peptide complexes and NKG2D receptor (Galazka et al., 2007). Tolerance to this autoimmune disease was induced by non-characterized peptide/Hsp70 complexes derived from the CNS of EAE mice. The activation of NK lymphocytes and the involvement of the NKG2D receptor and its ligand, H60, were the basis of the tolerogenic effect of the peptides complexed with Hsp70 (Galazka et al., 2006). A CNS-derived peptide, HINT138–57, which was administered in complexed form with Hsp70 by pretreatment, modulated NK cell-dependent immunity in animals sensitized to EAE. The immunoregulation mediated by NK lymphocytes was based on dual receptor stimulation (CD94 and NKG2D) on the membrane of NK lymphocytes (Galazka et al., 2014). This new mechanism of immunoregulation could deliver significant consequences for the control of autoimmune responses in the CNS and potential treatment strategies in autoimmunity and MS.

### 4.5 Granzymes and NK cells

Five granzymes are present in the human body: A, B, H, K, and M. The biological effects of granzyme A (GrA) and granzyme B (GrB) are comprehensively understood, but the biological role of the other granzymes is largely unknown. GrB causes rapid cell degeneration, mainly by activating caspases. GrA, on the other hand, causes cell death that does not depend on caspases (Boivin et al., 2009). NK cells cause an apoptotic process, independent of caspases, that involves NK cell degranulation and mitochondrial damage in activated T lymphocytes. GrA and granzyme K (GrK) can both cause this type of cell death; however, GrK is transferred to target cells more preferentially (Jiang et al., 2011). GrK causes cell death that does not depend on caspases and is very similar to GrA-induced cell death in terms of morphology and biochemistry, although GrA and GrK have very different substrate specificity fields (Hay and Slansky, 2022).

Studies involving granzyme transfer suggested that GrK was a critical factor in NK cell-mediated cytotoxicity in response to activated T lymphocytes. This agreed with the findings that GrK was expressed preferentially in CD56^*bright*^ in NK lymphocytes (Jiang et al., 2011). Cell death in activated T lymphocytes primarily resulted from mitochondrial apoptosis linked to the production of Reactive Oxygen Species (ROS). GrA and GrK were the main inducers of this type of death. There was a much greater level of expression of GrK in the CD56^*bright*^ NK cell subpopulation of patients who had received treatment (Peng et al., 2021). The findings of this study supported the function of GrK in modulating T-cell responses by CD56^*bright*^ NK cells. CD56^*bright*^ NK cells that regulate immunity eliminate autologous activated T cells using a perforin-dependent degranulation pathway that involves two related granzymes, GrK and GrA (Melsen et al., 2016). Also, granzyme B-mediated cell death was reported by Tr1 cells, which were comparable to CD56^*bright*^ NK cells, on other counts, including the secretion of IFN-gamma and IL-10 at the same time and their induction by IL-27 (Jiang et al., 2011).

CD56^*bright*^ NK cells were initially regarded as having no cytotoxic ability because they expressed much less perforin and almost no GrB in comparison to CD56^*dim*^ NK cells (Carrega et al., 2014). CD56^*bright*^ NK cells expressed levels of GrA that were comparable with CD56^*dim*^ NK cells, virtually all of which were positive for GrK, whereas CD56^*dim*^ NK cells (and almost all CD4^+^ T cells) did not express GrK.

## 5 Multiple sclerosis and natural killer cell

### 5.1 Genetic and non-genetic relationship between MS and NK cells

A systematic review thoroughly examined multiple susceptibility loci for multiple sclerosis and their genomic risks (Kemppinen et al., 2011). In addition to genes affecting adaptive immune cells, other genes also affect the innate immune system, including natural killer cells (Li et al., 2021). Consequently, gene variants involved in natural killer lymphocyte function are known to exist in multiple sclerosis. Cell surface glycoproteins such as CD226 appear on cytotoxic lymphocytes, including natural killers (Liu et al., 2017). The CD226 level is generally reduced in MS patients compared to the control group (Liu et al., 2017). In addition, CD226 plays a crucial part in NK cell-mediated T-cell elimination (Ardolino et al., 2011). Many MHC class II risk alleles are associated with MS (such as HLA-DRB1*15:01, HLA-DRB1*13:03, and HLA-DRB1*03:01), while MHC class I alleles may confer protective effects (such as HLA-A*02:01, HLA-B*44:02, and HLA-B*38:01) (Moutsianas et al., 2015).

The environment is considered a factor linked to MS; Vitamin D deficiency, infection with EBV and CMV, smoking, and obesity in adolescents are the most impactful and better understood among the factors related to environmental exposure (Beliën et al., 2022). Vitamin D maintains immune equilibrium by suppressing inflammatory cytokines produced by T lymphocytes (Smolders and Damoiseaux, 2011; Smolders et al., 2011). *In vitro*, cytotoxicity increases with 1,25-dihydroxy vitamin D treatment of NK cells without altering proliferation (Balogh et al., 1999). Adolescents and adults with EBV infection may develop infectious mononucleosis associated with multiple sclerosis (Cohen, 2000; Loosen et al., 2022). Natural killer cells are major players in the first line of immunity against EBV infection as they participate in innate immunity to the virus (Williams et al., 2005). Additionally, positive CMV serology indicates an increased MS risk, suggesting CMV contributes to disease development (Sundqvist et al., 2014). Tobacco use is associated with an increased risk of developing MS, possibly as a result of its inflammatory properties in the respiratory tracts, which can lead to systemic and chronic inflammatory responses. Smoking continues to play a role in MS pathology once the disease develops by increasing the frequency of relapses and reducing the time to progressive form (Ashare and Wetherill, 2018). The NK cells are affected by smoking in several ways, but an association between altered NK cells and MS currently can’t be established. Conversely, obesity negatively impacts the immunity (Faner et al., 2014; Gerriets and MacIver, 2014; Castoldi et al., 2016). Obesity and decreased physical activity independently increase the risk of developing MS. Obesity is associated with chronic inflammation that may be involved in the pathogenesis of MS. There is a lack of evidence regarding the relevance of these mechanisms to the development of MS (Varghese et al., 2019). Obesity also alters the NK cell population, demonstrating reduced cytotoxicity and cytokine production (Bähr et al., 2017). In this regard, it is impossible to establish a specific link between MS and obesity’s effect on NK cells. As a general rule, both smoking and obesity lead to pro-inflammatory conditions (Mimpen et al., 2020).

### 5.2 Involvement of different NK cell receptors in MS

NK cell activating and inhibiting receptors are a complex group of receptors that use opposing signaling patterns to enhance or suppress activation. Natural killer cells can eliminate abnormal cells, marked by high expression of stress-responsive ligands, enhancing activating interactions, and by decreased expression of HLA class I molecules, lowering the inhibition caused by HLA-specific inhibitory receptors (Schuster et al., 2014). Natural killer lymphocyte receptors on the surface consist of inhibiting as well as activating molecules, some expressing a variety of unpredictable, diverse, and overlapping forms. The interaction of these receptors on the surface with their corresponding substances controls the functionality of natural killers, and the combination of signals from these receptors determines the activation state of the natural killer lymphocyte (Pegram et al., 2011). Changes in natural killer lymphocyte counts and functionality have been associated with various human autoimmune disorders. For instance, reduced numbers of natural killer cells in the blood, decreased receptor expression, or decreased natural killer function have been seen among individuals suffering from type 1 diabetes and systemic lupus erythematosus (Kucuksezer et al., 2021).

There has been a new perspective on multiple sclerosis predisposition emerging since T cell receptors, and KIRs recognize HLA class I molecules in parallel. Since HLA-A3- restricted CD8^+^ T lymphocytes play an important role in its activation in a humanized mouse model, it is important to consider the role of natural killer lymphocyte receptors such as KIR3DL2, which are capable of detecting HLA-A3 (Crux and Elahi, 2017). Recently, we have gained further insight into the role of HLA-B and HLA-C alleles as KIR ligands in multiple sclerosis. There are a number of receptors that recognize HLA-Cw5, including KIR2DL1, KIR2DS1, and perhaps also KIR2DL2 and KIR2DL3 located on natural killer cells or a subpopulation of T lymphocytes, indicating that the HLA-KIR pathway may play a role in immune function modulation. There has been a similar protective effect noted for HLA-Bw4 ligands of KIR3DL1 (Jiao et al., 2008).

A family of genes known as CD94/NKG2 can be found on human chromosome 12 as part of the natural killer gene complex. Four NKG2 genes in humans (NKG2A, C, E, and F) were associated with the expression of the NK-activating receptors NKp30, NKp46, and CD94/NKG2D on the surface (Kaur et al., 2013). T-cell activation is reported to be regulated by CD94/NKG2A receptors in EAE. Using antibodies that block Qa1 NKG2A interactions, there is a reduction in cellular infiltrates and activation of microglia, as well as an alteration in the cytokine profile (namely, a reduction in IL-17 and IFN-γ and an increase in IL-4 and IL-10) of CD4^+^ T cells in the central nervous system (Chewning et al., 2007). Moreover, Qa1 interaction with CD94-NKG2A receptors on CD8^+^ T cells gives rise to an anti-suppressive response that weakens suppressive functions of CD8^+^ regulatory T cells; thereby, Qa1-NKG2A interaction disruption results in improved CD8 regulation and decreased EAE progression (Kim and Cantor, 2011; Kaur et al., 2013).

As part of the leukocyte receptor complex on chromosome 19q13.4, there are genes encoding leukocyte immunoglobulin-like receptors (LILRs, LIRs, or CD85) closely related to the KIR genes. Soluble LILRA3 poses a potential competitive characteristic since it contains no transmembrane and cytoplasmic regions. Those with progressive multiple sclerosis are reported to have a larger number of circulating LILRB1^+^ CD8^+^ T lymphocytes and LILRB1^+^ natural killer cells in comparison to patients with relapsing-remitting multiple sclerosis (Storm et al., 2021). During active relapse-remitting multiple sclerosis, there is a reduction in LILRB4 levels in monocytes in the blood. While it is unclear what its ligand is, LILRB4 is capable of inhibiting CD4^+^ T cell proliferation when expressed on antigen-presenting cells (Liu et al., 2020).

### 5.3 Mechanisms of immune regulation by natural killers in MS and EAE

Contact-dependent cytotoxicity is a crucial function of NK cells. During this process, cytolytic granules that contain proteases, granzymes, and perforins are released onto the targets through lytic synapses. Additionally, target cells are subjected to caspase-dependent apoptosis by stimulating natural cytotoxic receptors (NCRs) (Nielsen et al., 2012; Laroni et al., 2016; Gauthier et al., 2019). Natural killer cells are thought to prevent autoimmune disorders by reducing autoreactive adaptive immune responses. Mimpen et al. examined the significance of this ratio in the context of individuals with multiple sclerosis by analyzing natural killers and T lymphocyte subsets, in addition to their prognostic significance in terms of disease course (Mimpen et al., 2021). According to them, the CD56^*bright*^ NK lymphocyte subset is usually regarded as the regulating subset and has an inverse correlation with IL-17A^+^ CD4^+^ T cell percentages, but this concept seems to be contradicted by clinical findings (Poli et al., 2009). Activated T lymphocytes can be eliminated by both CD56^*dim*^ and CD56^*bright*^ NK cells. Research on natural killer cells in MS has primarily focused on CD56^*bright*^ natural killers over the past few years, but our findings indicate that CD56^*dim*^ NK cells have the potential to be of considerable significance (Mimpen et al., 2021).

MS is usually defined as a condition caused by autoimmune inflammation and involving CNS demyelination caused by autoreactive T cells (Thompson et al., 2018). Previously, studies have demonstrated that natural killer cells regulate adaptive immune reactions early in the process of an immune challenge in lymphoid organs. NK cells activated by cytokines possess potent lytic capacity against autologous CD4^+^ T cells and macrophages when they engage TRAIL receptors on their surfaces (Nielsen et al., 2012; Laroni et al., 2016). Schuster et al. defined an unexpected interaction involving natural killer cells and CD4^+^ T lymphocytes following chronic viral infections. This interaction occurs in a nonlymphoid tissue, specifically involving TRAIL-expressing NK cells. They demonstrated a novel and unexpected capacity of NK cells in the context of chronic viral infection. In one sense, effector CD4^+^ T lymphocytes are major components of the immune response to chronic viral infections; however, some subtypes of natural killer cells selectively eliminate effector CD4^+^ T cells, which in turn reduces autoimmune responses. Taken together, although NK cell-mediated deletion of CD4^+^ T cells prolongs the chronicity of infection, it also limits the development of autoimmunity in response to viral infections (Schuster et al., 2014). HLA-E detection at high levels in CNS plaques has been linked to increased inactivity and a reduction in NK cytotoxicity among MS patients (Morandi et al., 2013). Lünemann et al. analyzed CD56^*dim*^ CD56^*bright*^ subsets of NK cells and the activity of these cells in peripheral blood samples from relapsing-remitting multiple sclerosis patients and healthy donors. Their results indicated that cytokine-stimulated expansion under *in vitro* conditions, along with IFN-γ secretory capacity, was markedly impaired in CD56^*bright*^CD16^–^ NK cells. In contrast, CD56^*dim*^CD16^+^ NK subsets exhibited no such functional compromise. This selective dysfunction in CD56^*bright*^CD16^–^ cells was attributed to a cell-autonomous mechanism, as the defect persisted in isolated NK cell populations tested in a separate validation cohort comprising both MS patients and healthy controls. Certain activities of CD56^*bright*^ NK cells, including the expansion of natural killer cells in response to cytokines and IFN-γ release, are impaired in MS patients’ NK cells (Lünemann et al., 2011).

EAE represents a great animal model that describes how self-reactive CD4^+^ cells may contribute to MS. EAE is initiated through immunization with antigen-specific T cells for myelin oligodendrocyte glycoprotein (MOG) (Constantinescu et al., 2011). In patients suffering from multiple sclerosis and EAE models, Th17 cells induce disease (Komuczki et al., 2019; Wagner et al., 2020). It is hypothesized that natural killers contribute to the prevention of EAE by inhibiting Th17 responses by killing monocytes that infiltrate the CNS (Jiang et al., 2017).

### 5.4 Natural killer cells in therapies for MS

Despite the promising therapeutic properties of CD56^*bright*^ NK cells, there are no treatments available for disease based on this mechanism. Even though other drugs have not been developed to affect NK cells, they may be beneficial. Several drugs were discussed in connection with NK cells. One of the earliest treatments for MS was interferon-1b, a ligand that inhibits the activity of CD56 NK cells. The immunoregulatory effect of interferon-1b may be attributed in part to the reduction of cytotoxicity in CD56^*dim*^ NK cells (Kaur et al., 2013). Moreover, interferon-1b appears to alter the ratio between CD56^*bright*^ CD56^*dim*^ NK cells in peripheral blood by increasing the number of CD56^*bright*^ of lymphocytes (Poli et al., 2009).

Natalizumab prevents immune cells from migrating by blocking the very late antigen-4 (VLA-4). Natalizumab increases the number of circulating NK cells, possibly due to its decreased migration capacity to tissues, such as the central nervous system (Schlesinger and Bendas, 2015). A decrease in CD56^*bright*^ NK cells seems to be more significant than the depletion of T lymphocytes in the CNS, which is consistent with the favorable outcomes of natalizumab (Krumbholz et al., 2008). So far, NK cells have been suggested to have both positive and negative impacts on MS. According to studies of the past 40 years, MS progression is linked to a low NK cell function. Nevertheless, other research revealed a connection between NK cell activity and central nervous system pathologies, implying that the impact of NK lymphocytes on MS may vary depending on their subtype or mechanism of action (Mimpen et al., 2020; Kucuksezer et al., 2021).

Glatiramer acetate (GA) is a product consisting of four amino acids found within myelin basic protein (MBP) (Glu, Ala, Lys, Tyr) (Schrempf and Ziemssen, 2007). Originally, the substance was intended to induce EAE as a synthetic antigen. Studies in vitro indicate that glatiramer acetate promotes NK cell cytolytic activity, targeting immature and mature dendritic cells (DCs) and activating Th-1 cells (Wens et al., 2021). GA does not appear to enhance or alter NK cells directly, but they may play an essential role in clearing GA-altered DC fields (Maghazachi et al., 2016).

The fingolimod drug works by antagonistically opposing the effects of sphingosine-1-phosphate (S1P), which inhibits the release from lymph nodes of self-reactive lymphocytes (Groves et al., 2013). As a result of lymphocyte accumulation in the lymph nodes, fingolimod causes lymphopenia; however, Natural killer cells appear to be more resistant than B- and T-lymphocytes (Schwichtenberg et al., 2021). Fingolimod appears to affect the NK cell population (Kowarik et al., 2011). CD56^*bright*^ NK cells enrichment within the CSF may be responsible for at least some of the effects of fingolimod (Schwichtenberg et al., 2021).

Dimethyl Fumarate (DMF) is an efficient therapeutic agent for MS. However, its effect on the immune system remains unclear. There is evidence that the number of CD56^*bright*^ NK cells, have increased among MS patients who received DMF. Lymphopenia is a major concern for MS patients in those who use DMF as a treatment because of an increased risk of progressive multifocal leukoencephalopathy.

Multiple sclerosis treatment can affect natural killer cell numbers (Laroni and Uccelli, 2020). In an integrated analysis of *in vivo* clinical data and *in vitro* mechanistic studies derived from two phase II clinical trials involving 22 individuals with MS, Bielekova and colleagues proposed a model in which daclizumab exerts its therapeutic effects through an immunological cross-regulatory pathway linking innate and adaptive immunity. Their findings posit that this mechanism is mediated by immunoregulatory CD56^*bright*^ NK cells modulating T-cell activity, as evidenced by both trial-derived observations and controlled laboratory assays. Also, they found that daclizumab was able to expand CD56^–^positive NK cells, possibly contributing to its beneficial effects. Additionally, they improved their cytotoxic and regulatory abilities (Bielekova et al., 2006). Recent studies have shown that CD56^*bright*^ NK cells expand after daclizumab due to IL-2 binding to their intermediate receptor. Daclizumab does not target this receptor (Sheridan et al., 2011). In addition, some MS sufferers who receive daclizumab develop autoimmune encephalitis. A reduction in regulatory T lymphocytes may contribute to the withdrawal of the drug (Bianchi and Ciccarelli, 2019).

It is important to isolate immune subsets, as demonstrated by daclizumab and fingolimod. Therefore, the treatment propagates its beneficial effects and prevents unwanted ones. In a wide range of autoimmune diseases, NK cells have been identified as potential biomarkers and therapeutic targets (Gianchecchi et al., 2021). Research is currently being conducted to determine whether the levels of natural killers and their functional capacity are prognostic and indicative of treatment outcomes in MS. Nevertheless, further validation is required to provide a mechanistic explanation for the patterns observed (Mimpen et al., 2021).

## 6 Perspective findings on the NK and MS

As the earlier studies demonstrate, MS models can experience both protection and pathogenic impact from NK cells. Different functions of natural killers or a difference in the subset of targeted natural killer cells may have caused these divergent findings. Multiple mechanisms exist through which natural killer cells are capable of modulating self-reactive T lymphocytes by inhibition of these cells (Pallmer and Oxenius, 2016). To promote autoreactive T lymphocyte production, natural killers secrete substances to regulate native CD4^+^ T lymphocyte differentiation into Th1 or Th2 cells, thereby influencing autoimmune reactions (Poggi and Zocchi, 2014). natural killer cells secrete IFN-γ, which is thought to contribute to Th1 polarization. It could manifest as a subset of NK cells that express antigen-presenting machinery (such as MHC class II) and directly activate self-reactive T lymphocytes (Paul and Lal, 2017). In spite of this, cells may be interacting with auto-reactive T lymphocytes in multiple mechanisms, with direct control demonstrated recently on activated T cells by natural killer cells. Conversely, natural killer cells’ degeneration,’ which is characterized by the loss of the number or function of these cells (a reduction in cytotoxicity), is often reported in MS patients’ peripheral blood (Gross et al., 2016). In these settings, reduced cytotoxicity of natural killer cells can impair the restraint of immunopathogenic cells. In contrast, a decrease in circulating natural killer cells may indicate increased recruitment to inflammatory sites (Yang et al., 2021).

NK cells have an essential and influential part in the progression of MS. Some evidence suggests that removing or deactivating NK cells is beneficial to MS disease progression. In contrast, there has been some suggestion that more NK cells are beneficial to the condition (Ahmadi et al., 2022). The discrepancy can result from the fact that subsets of natural killers still need to be fully characterized. Increasing CD56^*bright*^ NK cells or reducing CD56^*dim*^ NK cells in MS has been demonstrated to result in successful treatment outcomes (Laroni et al., 2016). Thus, CD56^*bright*^ NK cells may modulate immune responses by increasing cytokine production associated with decreased adaptive immunity. CD56^*bright*^ NK cells have the potential to suppress the effects of autoreactive T lymphocytes, one of the primary causes of inflammation in MS. In this way, autoimmunity may be limited. On the other hand, the medication that increases CD56^*dim*^ NK cells can have adverse effects on the patient. However, further research is necessary to validate these findings and determine the mechanism of action (Ahmadi et al., 2022).

After observing favorable results in MS patients following treatment with daclizumab, a monoclonal antibody that blocks the alpha chain of IL-2 receptors (IL-2Ra; CD25), natural killer cells began to receive attention due to their IL-2 receptors (Giovannoni et al., 2014). Even so, the function of natural killer cells remains controversial, as demonstrated by studies demonstrating both their beneficial and detrimental effects in MS and experimental autoimmune encephalomyelitis (EAE) induced in rodents (Gandhi et al., 2010). Natural killer cells exhibit their functions by balancing their stimulatory and inhibitory receptors (Trachtenberg, 2009). Therefore, some studies have determined the natural killer cell receptor (NKR) expression profiles in multiple sclerosis and evaluated their contribution to the development of the disease. An initial study investigating how KIRs and their HLA ligands contribute to the progression of multiple sclerosis showed that patient HLA-Bw4 levels were significantly lower than those of healthy individuals, suggesting that HLA-Bw4 may protect against the development of MS (Lorentzen et al., 2009). The HLA-Bw4 ligand binds to inhibitory receptors on NK cells, thereby conferring NK cells’ functional proficiency in humans. According to a second study, the expression of KIR2DL2, an inhibitory receptor associated with infection by viruses, increased in people with multiple sclerosis afflicted with herpes simplex virus (HSV)-1 (Rizzo et al., 2012). Furthermore, NK cells expressing KIR2DL2 failed to control HSV-1 infection (Rizzo et al., 2016). Overall, the data suggest that KIRs and their ligands may play a significant role in MS. Yet, additional research is needed to understand their allelic variants’ functions in greater detail (Kucuksezer et al., 2021).

## 7 Conclusion

Available data indicate NK cells’ involvement in autoimmune diseases. An inflammatory environment promotes the migration of circulating NK cells into the central nervous system, influencing their function. NK cells release cytokines that attract and stimulate other immune cells, such as macrophages, neutrophils, and autoreactive T and B cells, contributing to inflammatory responses and tissue damage. Nevertheless, data suggests that specific subsets of NK cells can prevent autoimmune disorders by directly targeting autoreactive B and T cells. Furthermore, CD56^*dim*^ and CD56^*bright*^ NK cells can behave favorably or adversely under different conditions regarding similar autoimmune diseases. CD56^*dim*^ NK cells developed demyelination by activating auto-antibody and perforin from plasma cells and effector T cells, and CD56^*bright*^ NK cells inhibit demyelination by suppressing plasma cells and effector T cells.
